# Lack of association between intraoperative handoff of care and postoperative complications: a retrospective observational study

**DOI:** 10.1186/s12871-019-0858-8

**Published:** 2019-10-15

**Authors:** Vikas N. O’Reilly-Shah, Victoria G. Melanson, Cinnamon L. Sullivan, Craig S. Jabaley, Grant C. Lynde

**Affiliations:** Department of Anesthesiology, Emory University School of Medicine, 1364 Clifton Road, Atlanta, GA 30322 USA

**Keywords:** Patient safety, Communication, Patient handoff

## Abstract

**Background:**

The significance of intraoperative anesthesia handoffs on patient outcomes are unclear. One aspect differentiating the disparate results is the treatment of confounding factors, such as patient comorbidities and surgery time of day. We performed this study to quantify the significance of confounding variables on composite adverse events during intraoperative anesthesia handoffs.

**Methods:**

In this retrospective study, we analyzed data from the American College of Surgeons National Surgical Quality Improvement Project (ACS NSQIP). We examined the effects of intraoperative handoffs between anesthesia personnel. A total of 12,111 cases performed examined at two hospitals operated by a single healthcare system that were that included in the ACS NSQIP database performed. The presence of attending and anesthetist or resident handoffs, patient age, sex, body mass index, American Society of Anesthesiologists Physical Status (ASA-PS) classification, case length, surgical case complexity, and evening/weekend start time were measured.

**Results:**

A total of 2586 of all cases in the NSQIP dataset experienced a handoff during the case. When analyzed as a single variable, attending handoffs were associated with higher rates of adverse outcomes. However, once confounding variables were added into the analysis, attending handoffs and complete care transitions were no longer statistically significant.

**Conclusions:**

Inclusion of significant covariates is essential to fully understanding the impact provider handoffs have on patient outcomes. Case timing and lengthy case duration are more likely to result in both a handoff and an adverse event. The impact of handoffs on patient outcomes seen in the literature are likely due, in part, to how covariates were addressed.

## Background

Intraoperative transitions of anesthetic care, also referred to as handoffs or handovers, are a potential source of adverse events [1–3]. Handoffs represent both an opportunity for better-rested providers to heighten vigilance but also are a potential source of medical errors and heterogeneous clinical care [2, 3]. Examining their impact is important to identify opportunities to improve patient safety but potentially fraught by confounding. For example, surgical case length has been associated both with handoffs and adverse postoperative events [4, 5].

Previous large retrospective investigations exploring the association between handoffs and adverse clinical outcomes have found conflicting results, potentially owing to differences in care models or the analytic approach to confounders [6]. In a recent examination of retrospective data from over 300,000 anesthetics in Ontario, Canada, handoffs were associated with an increased risk of death, readmission, and major complications within 30 days [1]. In contrast, a prior examination of over 140,000 anesthetics within a large American university hospital, adjusted for case severity, duration, surgical complexity, and patient comorbidities, found no association between handoffs and adverse outcomes [7]. Effect estimates vary widely within other similar investigations [2, 8, 9].

We sought to further examine the association between handoffs of anesthetic care in our care team model and a composite measure of adverse postoperative outcomes using The American College of Surgeons National Surgical Quality Improvement Program (ACS NSQIP), as its definitions have been validated [10]. Measures included in the NSQIP composite outcome are: progressive or acute renal failure, cardiac arrest requiring cardiopulmonary resuscitation, stroke, any type of surgical site infection or sepsis, myocardial infarction, unplanned intubation, mechanical ventilation greater than 48 h, pneumonia, deep vein thrombosis, venous thromboembolism, urinary tract infection, or readmission within 30 days.

## Methods

This study was approved by the Emory University Institutional Review Board (IRB00108382) prior to data acquisition and analysis, including a waiver of written informed consent. Our data analysis plan, including variables to include as confounders, was included with our IRB submission. This manuscript was prepared in accordance with the Strengthening the Reporting of Observational Studies in Epidemiology (STROBE) statement and guidelines for improved reporting of observational studies and propensity score analyses [11].

### Setting

Data for the present work were drawn from two academic hospitals (Emory University Hospital and Emory University Hospital Midtown) within Emory Healthcare (EHC), which share a common anesthesia information management system (AIMS). AIMS and other electronic medical record data are consolidated nightly into the EHC Clinical Data Warehouse (CDW). At both sites, anesthetic care is delivered via the care-team model. For each surgical case, a combination of attending physician anesthesiologists, trainee physicians (i.e. residents and fellows), and non-physician anesthetists (i.e. certified registered nurse anesthetists and anesthesiologist assistants) provide care in concert. The attending physician anesthesiologist maintains responsibility for one or more concurrent patients, supervising a trainee physician or anesthetist who maintains continuous presence in the operating room. Attendings prescribe and medically direct the anesthetic plan. Only one attending anesthesiologist is responsible for the care of a patient at a time; handoffs may occur amongst attendings and/or non-attendings indicating a transfer of responsibility.

There is no mandatory handoff communication tool that is required from attending to attending. Anesthetists and residents at both hospitals are requested to complete a paper communications tool that is used for both provider and recovery room transfers of care. However, compliance is not mandatory, and no data exists to evaluate the frequency this communications tool is utilized.

### Data collection

Both studied EHC hospitals are participants in NSQIP, which entails collection and reporting of structured, high quality patient-level demographic and outcomes data by abstractors hired and trained specifically for this purpose. Data from January 2014 through December 2017 were combined with patient-level data from the EHC CDW for analysis. All cases reported to NSQIP utilizing intraoperative EMR were included in this analysis.

### NSQIP reporting

NSQIP reporting and sampling procedures are defined and monitored by the American College of Surgeons [12]. EHC reports both targeted and sampled data to NSQIP. Specific targeted surgeries, with 100% reporting of data from cases performed include: breast, colon, hepatobiliary, thyroid, and vascular. All other NSQIP reported cases are sampled utilizing an 8-day sampling technique where the first 40 cases performed in a rolling 8-day period are reported.

### Design

This study is a retrospective analysis of routinely collected clinical data in the anesthesia record combined with the aforementioned NSQIP data, which is also collected routinely (using the aforementioned case sampling strategy) as part of surgical quality improvement monitoring at our institution. Due to the retrospective nature of the planned work, no a priori power analysis or sample size calculation was performed; the research protocol called for inclusion of all NSQIP data available within the study period.

### Primary outcome

The primary outcome (dependent variables) for this study was a composite outcome of NSQIP postoperative occurrences. Specifically, any patient flagged as having any of the following occurrences was considered to have experienced the composite outcome: progressive or acute renal failure, cardiac arrest requiring cardiopulmonary resuscitation, stroke, any type of surgical site infection or sepsis, myocardial infarction, unplanned intubation, mechanical ventilation greater than 48 h, pneumonia, deep vein thrombosis, venous thromboembolism, urinary tract infection, or readmission within 30 days.

### Primary and secondary exposures

The primary exposure (independent variable) of interest was handoff at the attending level. This was defined as the presence of more than one attending recorded on the anesthetic record via the AIMS.

The secondary exposure of interest was a complete handover of care. This was defined as the presence of more than one attending and more than one non-attending documented on the electronic anesthetic record. At the studied institutions, breaks for non-attending providers are documented through a separate mechanism; as such, routine breaks were not considered when determining complete handovers.

### Independent variables

The independent variables included in the present analysis were patient age, sex, body mass index (BMI), ASA-PS classification, case length, surgical case complexity, and evening/weekend start time. Total case length was recorded in NSQIP; missing values were calculated as the difference between the routinely recorded anesthesia start and stop times. Surgical case complexity was defined as the quantity of American Society of Anesthesiologists Relative Value Guide base units ascribed to the case [13]. Evening/weekend start time was a binary determination based on surgery start time occurring (on Monday through Friday) prior to 7 A.M. or after 7 P.M., or occurring any time on a Saturday or Sunday. Normality was assessed via visual inspection of normalized quantile-quantile plots; case length and surgical complexity were logarithmically transformed to improve normality.

### Statistical analysis

Statistical analysis was performed in R v3.3.2 (R Core Team, Vienna, Austria) using the RStudio platform v1.1.423 (R Studio Team, Boston, MA) [14, 15]. Baseline characteristics were compared on a single variable basis between those exposed to a handoff and those not exposed. Chi-square test was used for sex, ASA-PS classification, evening/weekend case, surgical specialty, and the presence of a composite NSQIP adverse event. Wilcoxon rank-sum test was used for age, BMI, and case length.

The association of the composite outcome with the primary and the secondary exposures of interest was tested in multiple ways. In addition to chi-square analysis described above, a single variable logistic regression model (binomial logistic regression with one Y/dependent variable with two levels, one X/independent variable) was constructed to test the unadjusted association of the outcome with the exposure. Additionally, two multiple variable logistic regression models were constructed (binomial logistic regression with one Y/dependent variable with two levels, multiple X/independent variables). The simpler model contained the exposure of interest plus age, sex, and ASA-PS classification. The final model contained these independent variables plus case length, surgical case complexity, and evening/weekend start time.

## Results

**Fig. 1:**
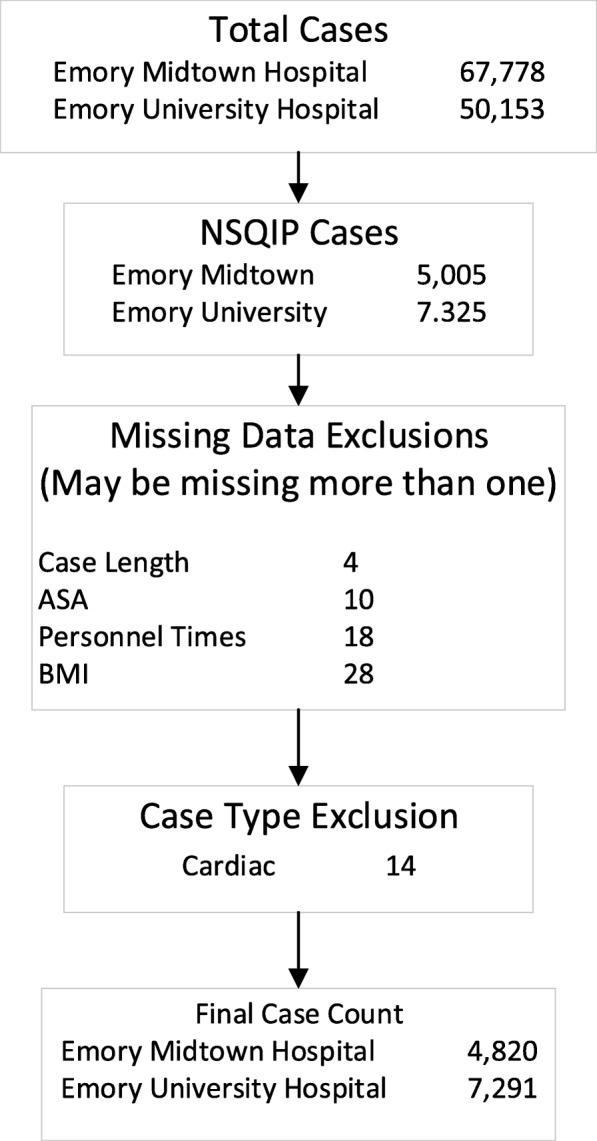
Diagram of cases

Following exclusions (Fig. 1), 12,111 (98.2%) of 12,330 available NSQIP cases were analyzed. As seen in Table 1, 2,586 (21.3%) were exposed to an attending handoff and 1320 (10.9%) were exposed to a complete handoff. This data represented the practice of 46 attending anesthesiologists, 76 anesthetists, and 52 residents or fellows. There were significant differences between the groups, including sex, ASA-PS classification, case length, BMI, and surgical specialty. In both cases, the single variable analysis showed that there was a significant increase in the rate of composite NSQIP adverse events amongst those exposed to either type of handoff. Due to these exploratory statistical differences in multiple baseline characteristics that were found on a single variable basis, further modeling was performed using binomial multivariable logistic regression to assess whether these potential associations with NSQIP adverse events were independently associated on a multivariable basis.

**Table 1:** Baseline characteristics of the study population

	Total	Attending Level Handoff			Complete Handoff		
		No Handoff	Handoff	*p*	No Handoff	Handoff	*p*
n (cases)	12111	9525 (78.7%)	2586 (21.3%)		10791 (89.1%)	1320 (10.9%)	
Age (mean (sd))	55.83 (15.42)	55.74 (15.51)	56.20 (15.10)	0.177	55.70 (15.45)	56.97 (15.12)	0.005
Male Sex (n (%))	4526 (37.4)	3531 (37.1)	995 (38.5)	0.192	3974 (36.8)	552 (41.8)	< 0.001
ASA Class (n (%))				< 0.001			< 0.001
1–2	4714 (38.9)	3847 (40.4)	867 (33.5)		4325 (40.1)	389 (29.5)	
3	6238 (51.5)	4808 (50.5)	1430 (55.3)		5466 (50.7)	772 (58.5)	
4–5	1159 (9.6)	870 (9.1)	289 (11.2)		1000 (9.3)	159 (12.0)	
Case Length (mean (sd))	155.65 (124.48)	131.15 (91.62)	245.91 (176.94)	< 0.001	138.72 (99.69)	294.10 (198.59)	< 0.001
Evening Or Weekend Case (n (%))	504 (4.2)	422 (4.4)	82 (3.2)	0.004	452 (4.2)	52 (3.9)	0.715
Body Mass Index (mean (sd))	29.64 (9.12)	29.72 (9.08)	29.33 (9.24)	0.056	29.71 (8.95)	29.05 (10.37)	0.012
SurgicalSpecialty (n (%))				< 0.001			< 0.001
ENT/OMFS	457 (3.8)	290 (3.0)	167 (6.5)		371 (3.4)	86 (6.5)	
Gastroenterology	6 (0.0)	6 (0.1)	0 (0.0)		6 (0.1)	0 (0.0)	
General/Oncology	6737 (55.6)	5328 (55.9)	1409 (54.5)		5961 (55.2)	776 (58.8)	
Neurosurgery	288 (2.4)	240 (2.5)	48 (1.9)		278 (2.6)	10 (0.8)	
Obstetrics/Gynecology	1307 (10.8)	961 (10.1)	346 (13.4)		1167 (10.8)	140 (10.6)	
Other	1943 (16.0)	1667 (17.5)	276 (10.7)		1833 (17.0)	110 (8.3)	
Thoracic/Pulmonary	135 (1.1)	119 (1.2)	16 (0.6)		125 (1.2)	10 (0.8)	
Urology	89 (0.7)	80 (0.8)	9 (0.3)		89 (0.8)	0 (0.0)	
Vascular	1149 (9.5)	834 (8.8)	315 (12.2)		961 (8.9)	188 (14.2)	
Composite NSQIP Adverse Event	1774 (14.6)	1268 (13.3)	506 (19.6)	< 0.001	1471 (13.6)	303 (23.0)	< 0.001

**Fig. 2:**
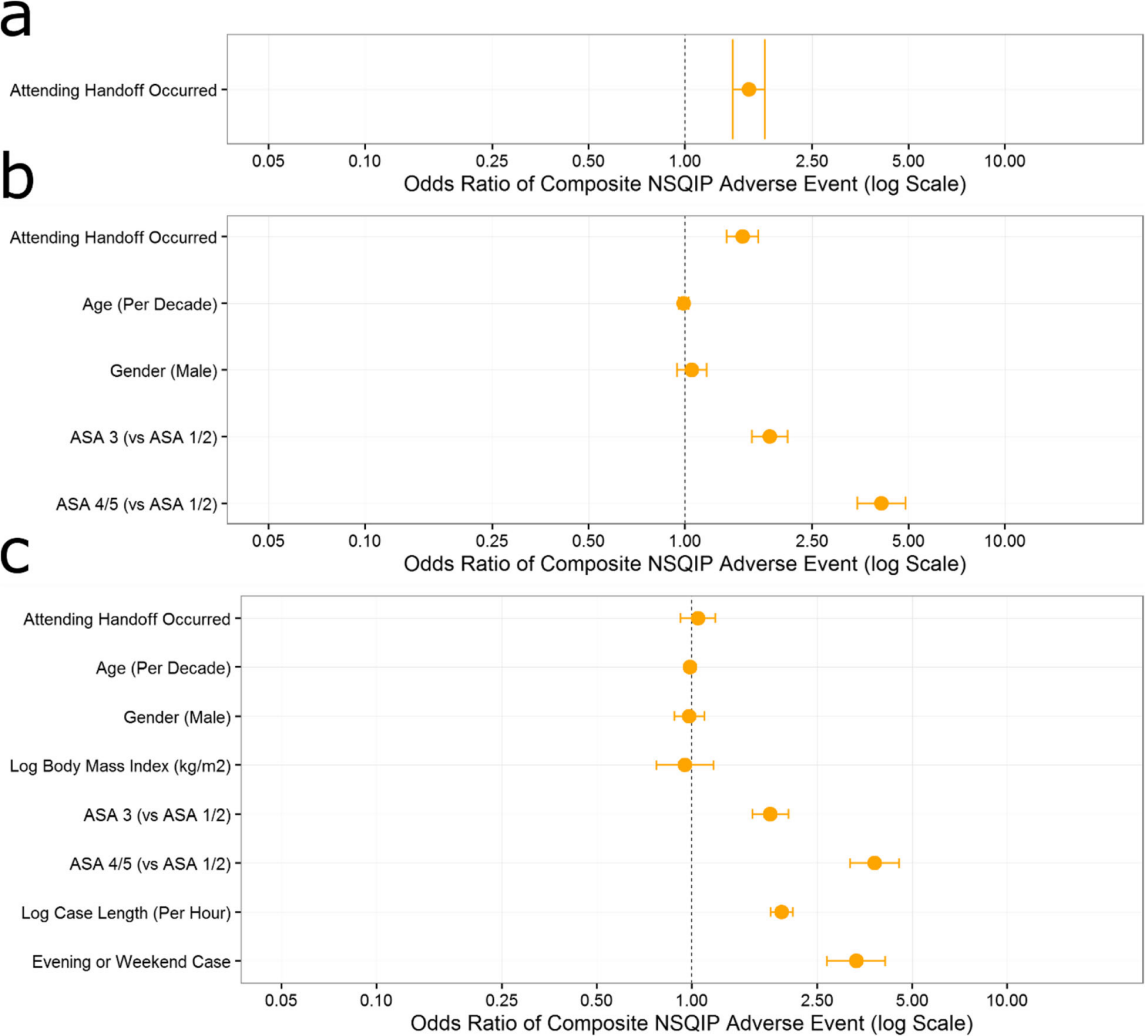
Complete results from **a** single variable, **b** four variable, and **c** six variable logistic regression analysis of the effect of attending handoff on the risk of experiencing a composite of NSQIP adverse events

**Fig. 3:**
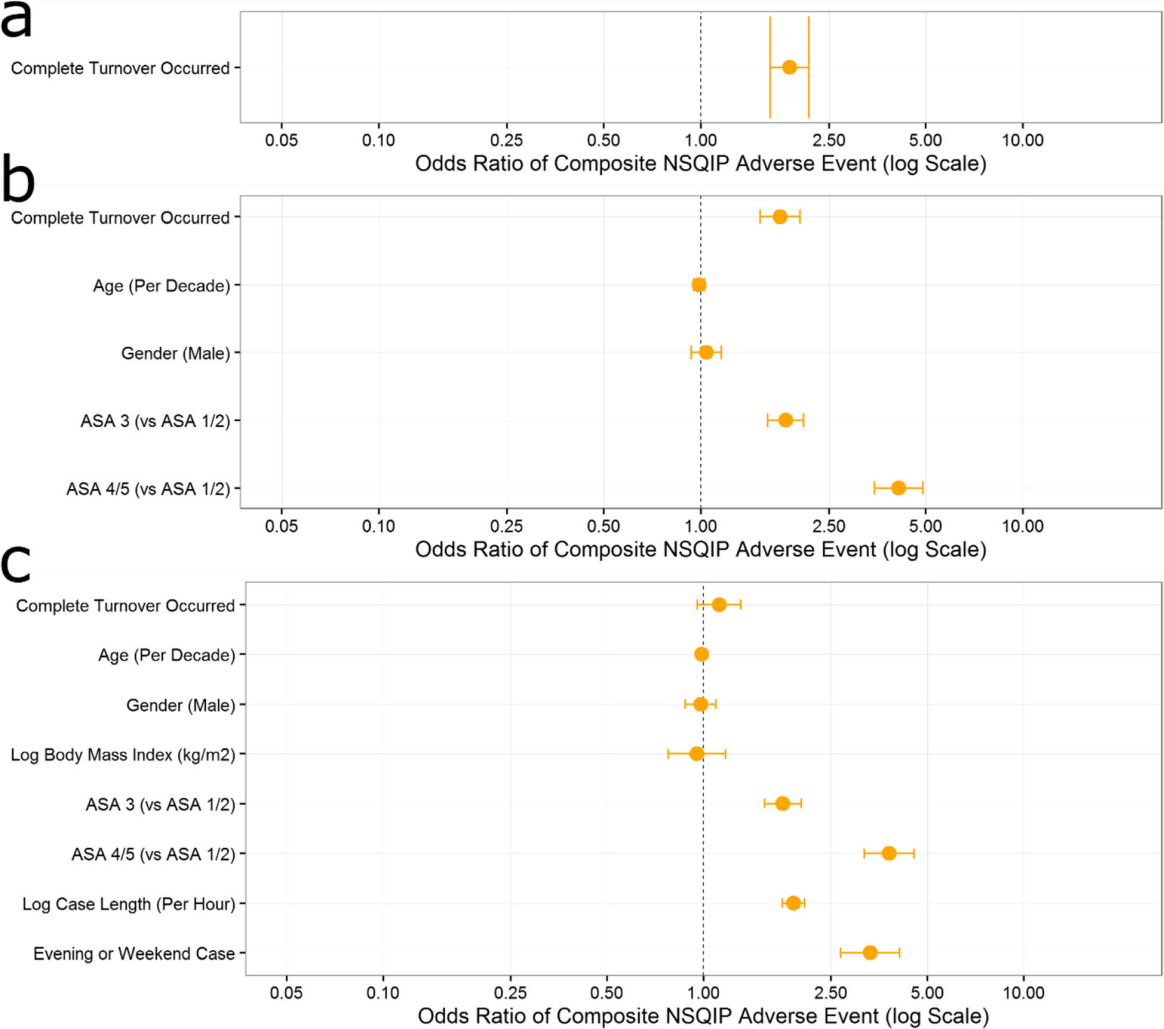
Complete results from **a** single variable, **b** four variable, and **c** six variable logistic regression analysis of the effect of complete handoff on the risk of experiencing a composite of NSQIP adverse events

On unadjusted logistic regression, an attending handoff was associated with an odds ratio (OR) of 1.58 (95% CI, 1.41 to 1.77) of an adverse event. Complete handoff was associated with an OR of 1.89 (95% CI, 1.64 to 2.17) of an adverse event. After adjusting for only the confounding variables of patient age, sex, and ASA-PS classification (1/2, 3, or 4/5), the OR for attending handoff and complete handoff decreased slightly (1.51 [95% CI, 1.35 to 1.70] and 1.76 [95% CI, 1.53 to 2.03], respectively) but remained significant. Added adjustment for case duration and evening or weekend case status resulted in the OR of an adverse event becoming nonsignificant for both attending handoff and complete handoff (1.05 [95% CI, 0.92 to 1.19] and 1.12 [95% CI, 0.96 to 1.31], respectively). The complete results from these models are presented in Figs. 2 and 3.

## Discussion

Our study demonstrated that anesthesia handoffs were associated with an increased rate of adverse events when examined in isolation. However, when accounting for all confounders, neither attending or complete handoffs of anesthesia care were associated with a composite measure of adverse postoperative outcomes. Comorbid conditions, case length, and case timing (i.e. evenings or weekends) were found to be important explanatory factors when examining the association between handoffs and adverse outcomes.

Given the complexity of perioperative care, attributing outcomes to any single intraoperative event, such as handoffs of anesthetic care, is problematic. Handoffs represent a potential entry point for error in patient care, and the Joint Commission estimates that 80% of serious medical errors involve failure of communication between caregivers [16, 17]. Changes in care team members may also introduce heterogeneity in clinical care. Conversely, handoffs allow for rested personnel to assume care, heighten vigilance and performance, and therefore possibly mitigate these risks. Estimates of handoff effects have varied widely in prior investigations; however, estimates of their effect in larger, more robust investigations have been modest [1, 2, 7, 8]. Effect estimates may therefore be influenced by sample size, case mix, and the relative balance of error introduction versus harm mitigation influenced by local practice.

Various structured tools have been proposed to mitigate the risk of communication lapses during handoffs [18, 19]. In our healthcare system, a structured communication tool is available for the providers in the operating room, however handoffs occurring between providers outside the operating room do not have readily available checklists. While various studies have demonstrated improved retention of elements of the patient’s care [20, 21], none have demonstrated differences in patient outcomes. One potential explanation for this is the immediate availability of the electronic medical record. Examining the patient’s medical record is frequently the most common task that an incoming provider performs [22], which mitigates communication lapses involving medication administration or pertinent past medical history. Another explanation could be the availability of structured communications tools, although implementation and use of these tools are highly context-dependent and with a great deal of potential variability in actual use.

Another potential explanation for the incongruity of our results with prior investigations may be that local practice patterns or experience surrounding handoffs and postoperative care can mitigate their potential harm. Our rates of attending-only and complete handoffs (21.3 and 10.9%, respectively) exceeded that of prior large investigations [1, 8, 9]. Differences in care team model, care team composition, or the conduct of handoffs may also influence their impact on clinical outcomes. Intraoperative handoffs may influence clinical outcomes by compromising the integrity of transitions to the post-anesthesia care unit (PACU) or intensive care unit through progressive knowledge loss. Our implementation of structured PACU handoffs or model of PACU care, with a dedicated attending anesthesiologist and trainee during daytime hours, may therefore mitigate the hypothetical negative impact of intraoperative handoffs.

Strengths of our study include congruence with the known literature. The explicitly performed analyses examining the impact of comorbid conditions, case length, and case timing, which are factors known to influence postoperative complications, yielded expected results. As noted by prior investigators, this type of investigation and analytic approach may be valuable means by which to assess the impact of local practice [9]. Similarly, the limited predictive ability of age and BMI in our model argue against overt residual confounding.

Our study has important limitations. Inherent to a retrospective, single-center study are the broad limitations that the work should be viewed as hypothesis-generating rather than hypothesis-testing, and that the results obtained from our center may not generalize to other contexts. In additionour sample size is based on the NSQIP sampling technique; although well-established, this may have served to introduce bias. NSQIP does not capture all potential clinical outcomes of interest, and not all outcomes are readily attributable to intraoperative anesthetic care. Surgical case volumes and types were heterogeneous, and although we attempted to adjust for surgical complexity, the strength of the observed association may vary within these categories. More broadly, residual confounding cannot be excluded despite appropriate sensitivity analyses. We conceptualized handoffs as being binary with respect to whether they occurred at the attending physician or entire anesthesia care team level. As such, our findings may not be comparable to prior studies utilizing stratified analyses of handoff counts. We did not account for the effect of staff breaks in our analysis because these may be inconsistently documented. While we utilize a structured handoff tool, we did not include this in our analysis because we were unable to assess utilization compliance. As previously discussed, local practice patterns regarding care team models and the conduct of handoffs are likely important but difficult to quantify, and therefore the generalizability of our findings to dissimilar settings is limited.

## Conclusions

Transitions of intraoperative anesthetic care are common, and controversy is likely to persist regarding their impact on clinical outcomes of interest. The balance between their potential to either introduce or mitigate harm may be heavily influenced by numerous elements of local practice that are difficult to quantify. Prior investigations suggest that intraoperative handoffs are associated with a sequential, nonzero risk for unintentional harm. However, the magnitude of these effect estimates has been variable. We posit that one significant source of variability in the literature is the accounting for confounders. Our investigation builds on prior work by examining the local impact of handoffs within the context of our care team model, conduct of care transitions, and approach to perioperative care. Additional work is needed to delineate how practice patterns or other factors, such as the use of structured communication tools, influence the impact of intraoperative anesthetic handoffs on postoperative outcomes.

## Data Availability

The datasets used and/or analyzed during the current study are available from the corresponding author on reasonable request. Public access to the NSQIP database is closed.

## Abbreviations

ACS NSQIP: American College of Surgeons National Surgical Quality Improvement Project
AIMS: Anesthesia information management system
ASA-PS: American Society of Anesthesiologists Physical Status
BMI: Body mass index BMI
CDW: Clinical Data Warehouse
EHC: Emory Healthcare
OR: Odds ratio
PACU: Post-anesthesia care unit
STROBE: Strengthening the Reporting of Observational Studies in Epidemiology
